# Thematic trends and knowledge structure on cognitive behavior therapy for insomnia: A bibliometric and visualization analysis

**DOI:** 10.3389/fpsyt.2022.940741

**Published:** 2022-09-15

**Authors:** Qianqian Xin, Dhirendra Paudel, Kai An, Youran Ye, Shuqiong Zheng, Lei Chen, Bin Zhang, Honglei Yin

**Affiliations:** ^1^Department of Psychiatry, Nanfang Hospital, Southern Medical University, Guangzhou, China; ^2^Guangdong-Hong Kong-Macao Greater Bay Area Center for Brain Science and Brain-Inspired Intelligence, Southern Medical University, Guangzhou, China; ^3^Mental Health and Yoga Pvt., Ltd., Pokhara, Nepal; ^4^Peking University Third Hospital, Beijing, China; ^5^School of Health Management, Southern Medical University, Guangzhou, China

**Keywords:** cognitive behavior therapy, insomnia, visualization, psychotherapy, bibliometric

## Abstract

**Objective:**

To find publications trend about cognitive behavior therapy for insomnia (CBTI) using bibliometric and visualization analysis. In this study, the authors sought to identify the publication trends of peer-reviewed articles about CBTI.

**Materials and methods:**

Analyses were focused on the past 18 years from 2004 to 2021. All searches were performed on the Web of Science Core Collection database. The search was repeated to include structural cognitive behavior therapy for insomnia. Quantitative analysis was assessed using the bibliometric tool. Visualization analysis was carried out using VOSviewer.

**Results:**

In the 736 articles reviewed, the number of publications has been increasing every year for the past 18 years. Behavioral sleep medicine and sleep were the most active journals published on CBTI. The United States and Canada had the highest scientific publications in the field. Morin CM and Espie CA were the most active authors. The study type mostly observed were randomized controlled trials, meta-analyses, and epidemiological. Publications on digital-based cognitive behavior therapy and accessibility to primary care settings represent the future trends of research on CBTI.

**Conclusion:**

Possible explanations for CBTI publication trends were discussed, including the emergence of the evidence-based therapy, feasibility, and scalability. Potential CBTI publications trends in the future and clinical implications were also discussed.

## Introduction

Cognitive Behavior Therapy for Insomnia (CBTI) is a psychotherapeutic approach to treating insomnia that includes cognitive, behavioral, and educational components (1). Cognitive content in CBTI attempts to change dysfunctional beliefs about sleep often termed cognitive restructuring. Behavioral content in CBTI consist of relaxation training (such as deep breathing exercises, progressive muscle relaxation, hypnosis, and/or meditation), stimulus control (changing the association of bedtime habit making sleep difficulties, like eating, watching TV, or using a cell phone or computer) (2), and sleep restriction (limits time spent in bed during the day and/or night to reestablish a consistent sleep schedule). Psychoeducation about the connection between feelings, thoughts, behaviors, and sleep is central to CBTI which is often aided by a sleep diary. Although CBTI lasts between 6–8 sessions (3), the length may vary according to the provider and person’s needs. Treatment may be as short as two sessions when given by a primary care provider (4), furthermore, there were reports of single-shot CBTI and its effectiveness in literature. Although cognitive therapy and behavioral therapy alone are effective, patients will experience the greatest benefit when all components of CBTI are combined with an 8-week course (5).

Insomnia is a major public health concern with a prevalence of about 10–20% (6). There are both pharmacological (such as benzodiazepines, z-drugs, melatonin, antidepressants, antipsychotics, and antihistamines) and non-pharmacological treatments (sleep hygiene, cognitive behavior therapy, relaxation therapy, multicomponent therapy, and paradoxical intention) available for insomnia (7). CBTI is one of the effective non-pharmacological treatments which can be applied to primary insomnia or comorbid insomnia in chronic conditions without the fear of drug interaction. However, due to the lack of trained clinicians, the uneven geographic distribution of knowledgeable professionals, and high costs, CBTI is an effective but underutilized treatment for insomnia (8).

Bibliometrics is a statistical method which provides researchers with qualitative and quantitative characteristics of literature by analyzing measurement indicators such as countries, journals, institutions, authors, and keywords, to describe current trends and discover the field hotspots. Bibliometric analysis has made a great contribution to the development of treatment and clinical guidelines (9). Therefore, this study intends to use bibliometrics to analyze the research status and research hotspots of CBTI, to lead the direction for related fields in the future.

## Materials and methods

The criteria for considering studies for this bibliometric analysis are described below.

### Study design

The papers published between the years 2004 and 2021 relevant to cognitive behavior therapy for insomnia were included. A detailed search strategy was developed for this study, and two researchers (QX and DP) in the field conducted article screening. Having more than one screener ensures the elimination of bias and errors in methodology resulting in data of high quality. The bibliometric and visualization analysis was done.

The current study aimed to examine possible trends in the volume of peer-reviewed publications on cognitive behavioral interventions for insomnia across time and to assess the evolution of such trends.

### Data source and retrieval strategies

**FIGURE 1:**
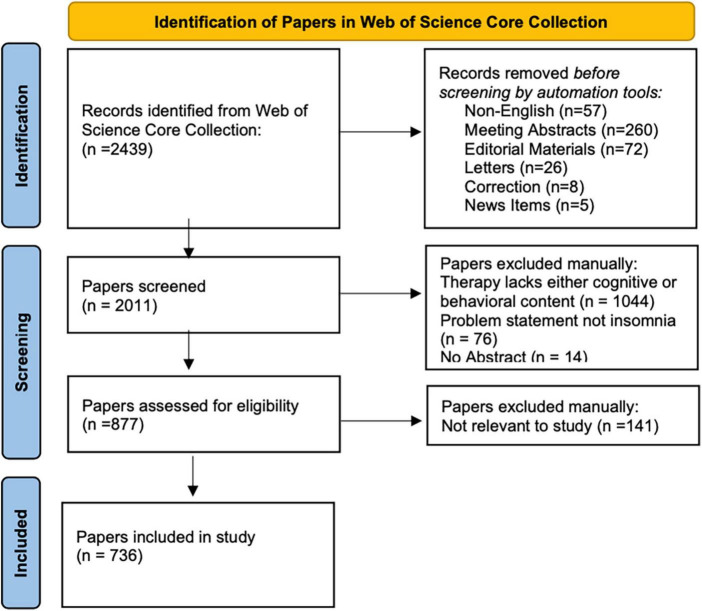
Flow diagram.

Data retrieval for this study was conducted on March 30, 2022. Data are drawn from the Web of Science Core Collection (WOSCC) using the Science Citation Index Expanded, the Social Science Citation Index, the Arts, and Humanities Citation Index, and the Emerging Sources Citation Index (SCIE, SSCI, AHCI, and ESCI) for the period of 18 years (2004–2021). Documents for analysis were restricted to original academic journal contributions (i.e., articles and reviews) which we will refer to as “papers.” Figure 1 shows the adapted Prisma flow diagram for the study (10).

After multiple iterations and using wildcards the following 2 concepts were used as topic search terms: concept 1, Cognitive Behavior Therapy (TS = (“cogniti* behavio* therap*”) OR Behavior Therapy (TS = (“behavio* therap*” OR “behavio* psychotherap*”) AND Cognitive Therapy (TS = (“cogniti* therap*” OR “cogniti* psychotherap*”) AND concept 2, insomnia (TS = (insomnia OR sleep Initiation and maintenance disorder* OR disorder* of initiating and maintaining sleep OR primary insomnia OR transient insomnia OR chronic insomnia OR secondary insomnia OR sleeplessness OR insomnia disorder* OR sleep wake disorder* OR sleep initiation dysfunction)).

### Eligibility criteria

Criteria for inclusion of a paper were as follows: (a) the main problem statement was insomnia (primary or comorbid), (b) the type of study was empirical, replication or review, and (c) the treatment condition used or main theme of the review was therapy consisting of both cognitive and behavioral in content.

As such the final included papers are relevant to cognitive behavior therapy for insomnia (CBTI). And those articles comparing behavior therapy and cognitive therapy for insomnia populated with boolean search strategies were excluded.

### Study variables

#### Dependent variable

Papers on Cognitive Behavior Therapy for insomnia.

#### Independent variable

Number of publications, distribution of journals, the contribution of authors, institutions, and countries, top-cited papers, and keyword co-occurrences.

### Quantitative and visualization tools

The Bibliometrix (11), an R package for bibliometric analysis was used to analyze the annual scientific productions, citation counting, journal, author, institution, and country distribution. The VOSviewer is visualization software that constructs a visualization network map from the co-occurrence of keywords (12).

## Results

### Number of publications

**FIGURE 2:**
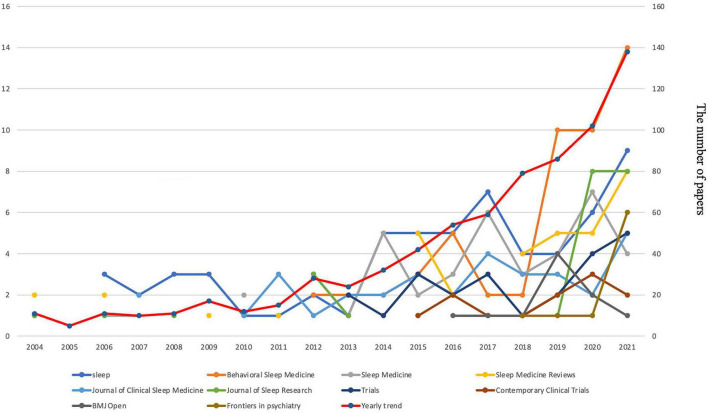
Yearly trends and top 10 journals publishing CBTI papers from 2004–2021.

The study included a total of 736 papers on CBTI. The number of annual publication papers increased every year, from 11 papers in 2004 to 138 in 2021. The study included papers on randomized clinical/controlled trials (RCT) (307), original research other than RCT (234), meta-analysis with systematic reviews (32), meta-analyses (19), systematic reviews (31) and reviews other than systematic reviews and meta-analysis (113). Figure 2 shows yearly trends and top 10 journals publishing CBTI papers from 2004 to 2021.

### Distribution of journals

Cognitive behavior therapy for insomnia papers were distributed in 236 journals. Sleep published the most papers (62/736, 8.42%), followed by Behavioral Sleep Medicine (52/736, 7.07%). The highest impact factors (IF) and h-index were observed with Sleep (IF: 5.849, h-index: 35) and Sleep Medicine Reviews (IF:11.609, h-index:21) (Table 1). Table 1 listed the top 10 journals according to volumes of publications on CBTI.

**TABLE 1:** Top 10 journals publishing papers on CBTI.

Rank	Journal	*n* (%)*	IF	Mean IF**	H-index
1	Sleep	62 (8.42)	5.849	5.0566	35
2	Behavioral Sleep Medicine	52 (7.07)	2.964	2.7614	13
3	Sleep Medicine	42 (5.71)	3.492	3.3352	17
4	Sleep Medicine Reviews	37 (5.03)	11.609	10.2598	21
5	Journal of Clinical Sleep Medicine	33 (4.48)	4.062	3.5858	17
6	Journal of Sleep Research	26 (3.53)	3.981	3.5456	11
7	Trials	23 (3.13)	2.279	2.0346	8
8	Contemporary Clinical Trials	12 (1.63)	2.226	2.2182	5
9	BMJ Open	10 (1.36)	2.692	2.4692	4
10	Frontiers in Psychiatry	9 (1.22)	4.157	3.3112	1

*Denominator = Total number of papers on CBTI retrieved from Web of Science Core Collection from 2004 to 2021 (*N* = 736); **Mean IF = Five-year mean impact factor for 2017–2021.

### Contribution of authors, institutions, and countries

There were 957 institutions that participated in the CBTI papers. Table 2 presented the top 10 institutions that contributed papers on CBTI. The University of Laval published the most papers (79/736, 10.73%), followed by the University of Pennsylvania (68/736, 9.24%) (Table 3).

**TABLE 2:** Top 10 authors in terms of publications on CBTI.

Rank	Author	Country	*n* (%)*	Citations	Average citations**	H-index
1	Morin CM	Canada	38 (5.16)	801	21.08	19
2	Espie CA	United Kingdom	34 (4.62)	674	19.82	21
3	Ritterband LM	United States	22 (2.99)	418	19.00	11
4	Savard J	Canada	22 (2.99)	359	16.32	13
5	Garland SN	Canada	18 (2.45)	114	6.33	11
6	Manber R	United States	18 (2.45)	276	15.33	15
7	Perlis ML	United States	18 (2.45)	233	12.94	14
8	Lack L	Australia	17 (2.31)	86	5.06	9
9	Van Straten A	Netherlands	17 (2.31)	223	13.12	10
10	Edinger JD	United States	16 (2.17)	512	32.00	14

*Denominator = Total number of papers on CBTI retrieved from Web of Science Core Collection from 2004 to 2021 (*N* = 736); **Average citations = Citation number divided by the number of journals publishing papers.

**TABLE 3:** Top 10 countries and institutions contributing papers on CBTI.

Ranks	Country	*n* (%)*	Institutions	Country	*n* (%)*
1	United States	296 (40.22)	University of Laval	Canada	79 (10.73)
2	Canada	62 (8.42)	University of Pennsylvania	United States	68 (9.24)
3	Australia	61 (8.29)	University of Oxford	United Kingdom	63 (8.56)
4	China	53 (7.20)	Karolinska Institute	Sweden	56 (7.61)
5	United Kingdom	52 (7.07)	Vrije University Amsterdam	Holland	51 (6.93)
6	Netherlands	34 (4.62)	University of Sydney	Australia	49 (6.66)
7	Sweden	30 (4.08)	Flinders University	Australia	39 (5.30)
8	Germany	27 (3.67)	University of Rochester	United States	37 (5.03)
9	Japan	19 (2.58)	University of Michigan	United States	36 (4.89)
10	Korea	18 (2.45)	University of Pittsburgh	United States	36 (4.89)

*Denominator = Total number of papers on CBTI retrieved from Web of Science Core Collection from 2004 to 2021 (*N* = 736).

Only 33 countries around the globe contributed to papers on CBTI. Table 3 shows the top 10 countries that published papers on CBTI. The United States published the most articles (296/736,40.22%), followed by Canada (62/736,8.42%) (Table 3).

The study identified 2,783 authors contributing to CBTI papers. Table 2 presented the top 10 authors with the highest yields of CBTI studies. Morin CM had the most papers (38/736, 5.16%), followed by Espie CA (34/736, 4.62%). Espie CA and Morin CM had the highest h-index of 21 and 19, respectively (Table 2).

### Top cited papers

Out of the top 10 cited papers in this study (Table 4), four were practical recommendations for the management of insomnia. One was a pilot study done on depression co-morbidity. One was a review on the relationship between sleep and pain. Two were meta-analyses on cognitive behavior therapy for insomnia. And two were a randomized controlled trial on CBT vs. z-drug (viz Zopiclone and Zolpidem) for insomnia.

**TABLE 4:** Top 10 most cited papers on CBTI during study period.

Rank	Title	Author	Journal	Year	Citations
1	Psychological and behavioral treatment of insomnia:update of the recent evidence (1998–2004)	Morin CM	Sleep	2006	749
2	Management of Chronic Insomnia Disorder in Adults: A Clinical Practice Guideline From the American College of Physicians	Qaseem A	Ann Intern Med	2016	663
3	How do sleep disturbance and chronic pain inter-relate? Insights from the longitudinal and cognitive-behavioral clinical trials literature	Smith MT	Sleep Med Rev	2004	542
4	Cognitive behavioral therapy for insomnia enhances depression outcome in patients with comorbid major depressive disorder and insomnia	Manber R	Sleep	2008	521
5	Practice parameters for the psychological and behavioral treatment of insomnia: An update. An American Academy of Sleep Medicine Report	Morgenthaler T	Sleep	2006	496
6	Cognitive behavioral therapy, singly and combined with medication, for persistent insomnia: a randomized controlled trial	Morin CM	Jama-J Am Med Assoc	2009	414
7	Cognitive Behavioral Therapy for Chronic Insomnia: A Systematic Review and Meta-analysis	Trauer JM	Ann Intern Med	2015	401
8	Dealing with sleep problems during home confinement due to the COVID-19 outbreak: Practical recommendations from a task force of the European CBT-I Academy	Altena E	J Sleep Res	2020	348
9	Cognitive behavioral therapy vs. zopiclone for treatment of chronic primary insomnia in older adults: a randomized controlled trial	Sivertsen B	Jama-J Am Med Assoc	2006	342
10	Comparative meta-analysis of behavioral interventions for insomnia and their efficacy in middle-aged adults and in older adults 55+years of age	Irwin MR	Health Psychol	2006	322

### Keywords co-occurrence and visual analysis

**FIGURE 3:**
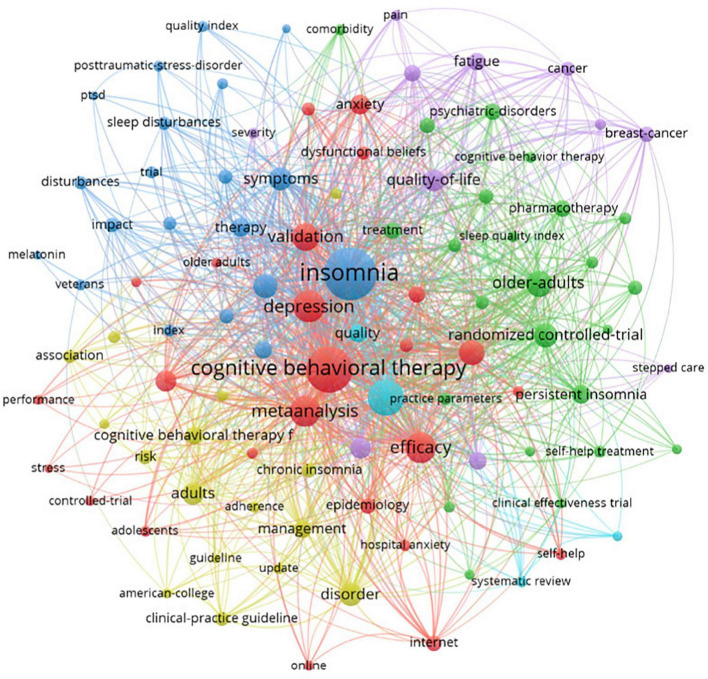
Network visualization map of co-occurrence on top 100 keywords. Each node is represented by a circle with labels for different words. Larger circles represent more frequently used words. The thickness and length of the lines linked between nodes represent the strength of the association between the corresponding nodes.

The research directions of CBTI publications collected by WoS were divided into five clusters (Figure 3). The top ten keywords as detected by VOSviewer were insomnia (*n* = 460), cognitive behavioral therapy (*n* = 358), sleep (*n* = 227), depression (*n* = 174), meta analysis (*n* = 167), efficacy (*n* = 161), validation (*n* = 131), older-adults (*n* = 121), severity index (*n* = 114) and randomized controlled trial (*n* = 102).

The visual analysis shows the research trends. Most of the participants were adolescents, adults, older adults, veterans, and women. The study type mostly observed were randomized controlled trials, meta-analyses, and epidemiological. CBTI has been used in comorbid conditions both physical and psychiatric conditions such as stress, post-traumatic stress disorder, anxiety, depression, pain, fatigue, and cancer. It can be drawn that measurement tools used to test the effectiveness of CBTI were insomnia severity index, sleep quality index, hospital anxiety depression scale, quality of life, polysomnography, and actigraphy. The appearance of words like the internet and online shows the digital adoption of cognitive behavior therapy in recent years.

### Excluded papers

Most of the papers excluded were about Cognitive behavior therapy for other disorders, notably CBT for tinnitus, cancer, pain, hypertension, and psychiatric disorders. The pharmacological management articles were also excluded.

## Discussion

### Global trend on cognitive behavior therapy for insomnia

This study found that the number of annual publication papers on CBTI increased every year. Sleep and Behavioral Sleep Medicine were the top journals of CBTI publications, among the top 10 authors who published CBTI papers, four are from the United States and three are from Canada. The University of Laval and the University of Pennsylvania were the top institutions that published papers on CBTI, and the United States and Canada were the top countries that published papers on CBTI.

This study found that the authors in an institution from countries in North America and Europe contribute more to CBTI research. China, Japan, and Korea taking a lead in Asian Continent. In the identified publications, the United States, Canada, Australia, and China were currently leaders in CBTI. And the study found that, among the top 10 authors who published CBTI papers, four are from the United States and three are from Canada. Four of the top 10 institutions on CBTI papers were from the United States, and another five were from Canada, Sweden, the United Kingdom, Australia, and Holland. This might be due to the better economic and scientific status of each country or territory and better communication among them similar to another bibliometric analysis study (13).

The changes in the number of scholarly publications in a field were an important indicator of trends in the field (14). The journal with higher publication frequency provides researchers with guidelines for paper publication. The study found that Sleep and Behavioral Sleep Medicine had a high frequency of CBTI publications. Nowadays, researchers are probing on metabolic, hormonal, genetic, cellular, and subcellular effects of sleep disturbances (15). However, CBTI research is still in the early stage, and the cooperation between authors is still limited. Facilitating collaboration between authors, institutions and countries will increase the number of authors who regularly publish in the field and contribute to a more effective exchange of experiences on CBTI research to improve the quality of treatment methods for insomnia.

### Hot topics on cognitive behavior therapy for insomnia

The study found that CBTI has been widely used in comorbid conditions both physical and psychiatric conditions such as stress, post-traumatic stress disorder, anxiety, depression, pain, fatigue, and cancer. Co-occurrence can be drawn that the study type mostly observed were randomized controlled trials, meta-analyses, and epidemiological, and measurement tools used to test the effectiveness of CBTI were insomnia severity index, sleep quality index, polysomnography among others.

Cognitive behavior therapy for insomnia has been accepted as a first-line treatment, and American Academy of Sleep Medicine (AASM) guidelines also recommend CBTI as standard psychological and behavioral therapy for chronic insomnia. In addition, The National Institute for Health and Care Excellence (NICE) guidelines and the latest European Sleep Research Association guidelines also recommend CBTI as an initial treatment for chronic insomnia in any age group (16). Numerous previous studies have shown that CBTIs were effective in reducing the severity of insomnia, also in primary care settings (17). Results from previous studies on the effectiveness of CBTI have shown response rates over 60%, with good initial responses maintained after 6 months (18). Silversten et al. compared CBTI, Zopiclone (a drug treatment), and placebo in a sample of 46 older adults diagnosed with chronic insomnia. They found that over just 6 weeks, patients who received CBTI reported more improvements in sleep efficiency, slow-wave sleep, middle of the night awakenings, compared with patients who received the drug or a placebo. These results persisted even at 6 months, with patients receiving CBTI reporting greater improvements in sleep efficiency than those receiving Zopiclone (19).

Cognitive behavior therapy for insomnia also has a good effect on insomnia with comorbid conditions, especially depression and anxiety. Anxiety disorders are the most common mental health disorder worldwide, with a prevalence of 25%, and people with anxiety disorders often experience poor sleep quality, and further exacerbate anxiety. Molecular imaging evidence also suggests that there are specific neurotransmitter mechanisms of sleep-wake regulation associated with anxiety (20). It is known that there is a benefit of CBTI not only for insomnia but also for comorbid anxiety disorder (20). A report of ten studies examining the effect of CBTI on depressive outcomes in patients with co-occurring depression and insomnia reported that CBTI may be an appropriate stand-alone treatment for co-occurring depression and insomnia, or in combination with antidepressants (21). Several other meta-analyses have similar results (22). Apart from anxiety and depression, CBTI has been applied to the treatment of comorbid insomnia in pain, migraine, fatigue, chronic obstructive pulmonary disease, asthma, Parkinson’s disease, and other neurological as well as psychiatric disorders (23).

Subjective-objective sleep differences have long been a difficult problem in sleep medicine practice, which is because sleep is a subjective perceptual experience of a person, so subjective sleep parameters are often inconsistent with objective sleep parameters measured by polysomnography or actigraphy. The measurement tools or indicators like polysomnography, actigraphy, and insomnia severity index (ISI) are being widely used in the field of sleep (24). Polysomnography was considered the “gold standard” for assessing sleep characteristics and stages. An advantage of actigraphy is that it can assess sleep outcomes over multiple nights, allowing an assessment of sleep patterns and variability (25). ISI is particularly useful in population and clinical settings where more than one condition needs to be measured at a time without overburdening the patient (26). With the help of psychometric tools and technologies available, studies have found that CBTI is effective regardless of whether the subjective-objective sleep differences are large or small (27).

### Most potential areas of cognitive behavior therapy for insomnia

With keywords co-occurrence, it can be drawn that digital CBTI is a research hotspot in recent years. CBTI is recommended as a first-line treatment. However, some doctors were difficult to carry out CBTI due to a lack of professional knowledge, sufficient time, qualified psychologists, high cost, and other reasons (28).

The number of trained CBTI practitioners is scarce. Most patients learn about sleeping tablets than CBTI due to prescribing physicians and advertisements and misconceptions about CBTI (8). Health care workers and physicians play an important role in the dissemination of CBTI (8). In addition, there is an optimistic view regarding CBTI utilization. Even primary healthcare workers can deliver 6-session CBT to insomnia patients without the involvement of physicians and with minimal supervision (29). Furthermore, reports showed that brief CBT can be delivered with teleconsultation and self-help materials (30). These findings suggest that brief and effective CBTI can be developed to suit primary care settings. In primary care settings, with limited resources, various modifications of CBTI have been investigated such as group CBTI, and brief CBTI (4). Several CBTI modifications are effective and comparative to standard CBTI in routine care settings (31).

Furthermore, the application of digital CBTI may bridge the gap. Digital CBTI can be divided into different types including internet, phone, email, and mobile app-based CBTI. Compared with face-to-face CBTI, online CBTI is a cost-effective and time-effective, scalable, and accessible, meanwhile interactive web design, animation technology, automated multimedia web applications coupled with effective clinical support can greatly improve patient motivation and outcomes (32). Espied et al. (33) found that the online CBT was related to adherence to some therapists’ guidance in patients with co-morbid insomnia and depression. The results of sleep onset latency and sleep efficiency of verbally contacted telephone-based CBTI were suboptimal, possibly because therapist-patient interaction may play a key role in the success of digital CBTI. Online CBTI with therapists significantly improved several sleep metrics, including self-reported total sleep time (compared to phone-based CBTI), wake after sleep onset (compared to mobile app-based CBTI, phone-based CBTI, and web-based CBTI without a therapist), and sleep efficiency (compared to online CBTI without a therapist). In addition, a meta-analysis of assessment methods analyzing digital CBTI found that compared with face-to-face therapy, digital CBTI prolonged self-reported total sleep time, shortened sleep onset latency, shortened wake after sleep onset, and better sleep efficiency (32). Online CBTI combined with virtual therapists is preferred considering better treatment outcomes, offering promising results, scalability, and addressing the lack of real therapists in the future (32). At the same time, future research should focus on raising the awareness of primary care providers (general practitioners, primary care nurses, etc.) about CBTI, optimizing CBTI online courses (for example, doing some preparation before each CBTI meeting, having a specific agenda, and schedule the meeting at a time when the primary care provider is less tiring), increased training of primary care providers (such as more thorough discussion of the different components of CBTI treatment, negotiate with the patient on the goals of each session, regular phone calls to actively follow up with the patient) and providing patients with some relevant paper materials (34), and explore other new behavioral treatments such as population-specific CBTI or combination therapy. Digital CBTI will help provide immediate evidence-based care to individuals in rural/remote areas during the current COVID-19 pandemic and social distancing in the future (35).

### Limitation

There are some limitations of our bibliometric study. Firstly, the data was searched from the database of WOSCC and only papers published in the English language were selected. The strategy might have missed some important literature in the field. Secondly, the citation of the same paper in different databases is different, and the citation of WOSCC is different than that of google scholar. Although the variable contributions of authors include co-authors, the possibility of co-authorship and their contribution to the field could not be ascertained. These issues should be addressed in future studies.

## Conclusion

This research systematically and comprehensively analyzes the research characteristics and trends of the CBTI paper. The field of CBTI is maturing, with great potential and broad prospects. The quality of research in this field is high. The top journals can be used by researchers as target journals for publications on CBTI. Additionally, the topics of special interest and novel studies in this field are indicative of the future directions in CBTI research. Over the past 18 years, CBTI has dominated the professional literature. CBTI was applied to insomnia as well as comorbid conditions with insomnia. New approaches like digital-based CBTI have emerged, likely influenced by the increase in the use of digital platforms. Future research should focus on creating new delivery models for CBTI that emphasize the prevention of insomnia and the scalability of treatments.

## Data availability statement

The raw data supporting the conclusions of this article will be made available by the authors, without undue reservation.

## Author contributions

QX and DP worked with KA to formulate the search formula, identify the search library, literature search, and data analysis. LC, YY, and SZ helped draft the manuscript and screen the data. DP visualized and revised the manuscript. HY and BZ helped with the conception and revision of the full text. All authors contributed to the article and approved the submitted version.
